# Water, Sanitation, and Hygiene Infrastructure and Resources in Schools in Belize during the COVID-19 Pandemic, 2021–2023

**DOI:** 10.3390/ijerph21040470

**Published:** 2024-04-12

**Authors:** Anh N. Ly, Kelsey McDavid, Christina Craig, Dian Maheia, Yolanda Gongora, Alexandra Medley, Francis Morey, Russell Manzanero, Gerhaldine Morazan, Allison Lino, Vickie Romero, Rosalva Blanco, Kanako Ishida, Matthew Lozier, Kristy O. Murray

**Affiliations:** 1 Department of Pediatrics, National School of Tropical Medicine, Baylor College of Medicine and Texas Children’s Hospital, 1102 Bates Ave, Houston, TX 77030, USA; 2 Division of Foodborne, Waterborne, and Environmental Diseases, Centers for Disease Control and Prevention, 1600 Clifton Rd, Atlanta, GA 30333, USA; 3 Belize Ministry of Education, Culture, Science, and Technology, West Block Independence Plaza, Belmopan, Belize; 4 Belize Ministry of Health and Wellness, East Block Building, National Assembly, Bliss Parade, Belmopan, Belize; 5 United States Public Health Service, 1101 Wootton Parkway, Suite 300, Rockville, MD 20852, USA

**Keywords:** WASH in schools, hand hygiene, COVID-19, Belize

## Abstract

Access to water, sanitation, and hygiene (WASH) resources in schools is critical for disease prevention and control, especially during public health emergencies. In Belize, systematic, national data on WASH in schools are needed to inform public health decisions and interventions. From December 2021 to January 2022, a national survey was sent electronically to government and government-aided primary and secondary schools in Belize (N = 308) to gather information on WASH services. From the survey, 12 pilot schools were selected based on the highest self-reported need for WASH resources to participate in additional evaluation and intervention, which included environmental nudges, supplemental supply provision, and hand hygiene education. To understand how the progression of the COVID-19 pandemic may have influenced hand hygiene, facility assessments to evaluate access to hand hygiene resources were conducted in person when most schools reopened for face-to-face learning during the pandemic (March 2022) and 15 months later (June 2023). Among the schools participating in the national survey (N = 221), 55% reported times when water was not available at the schools. Almost 9 in 10 schools (89%) had a functional handwashing station, and 47% reported always having soap for handwashing. Between baseline and follow-up at the 12 pilot schools, we observed decreases in the proportion of functional handwashing access points (−11%), functional handwashing access points accessible for individuals with disabilities (−17%) and small children (−29%), and functional alcohol-based hand rub dispensers (−13%). Despite the ongoing COVID-19 pandemic, we observed gaps in WASH resources in schools in Belize during the onsite assessments at the pilot schools. Schools should be encouraged and provided with WASH resources to maintain vigilance for disease control measures.

## 1. Introduction

Diarrheal diseases remain a leading cause of death in low- and middle-income countries (LMICs), especially among children [1,2]. In 2019, approximately 69% of diarrheal diseases could be attributed to inadequate water, sanitation, and hygiene (WASH) [3]. WASH infrastructure and resources are critical in school settings since children spend a substantial amount of time at school annually. The United Nations Sustainable Development Goal (SDG) 6.2 aims to achieve sanitation and hygiene for all, while SDG 4.A aims to provide a safe learning environment for all [4,5]. Despite the ongoing global efforts to advance and improve access to WASH services, there are remaining gaps in achieving these goals. As of 2021, only 58% of schools globally had basic hygiene services, which is defined as access to a handwashing facility with soap and water [6]. The latest Joint Monitoring Program (JMP) data from Latin America and the Caribbean in 2019 showed that only 59% of schools had access to basic hygiene services [6]. 

During the COVID-19 pandemic, the World Health Organization (WHO) and the U.S. Centers for Disease Control and Prevention (CDC) recommended hand hygiene as a key preventive public health measure to minimize the spread of infections [7,8]. However, many countries, including Belize, still lacked data on the availability of WASH infrastructure for local governments to recommend and enforce public health measures related to WASH. Bordering Guatemala and Mexico, Belize is a small Central American country with stark disparities in poverty. Nationally, 35.7% of the population is in poverty, but it can reach as high as 70% in rural areas [9]. In Belize, 1 in 3 schools have no or limited access to hygiene services [10]. To our knowledge, limited national-level school WASH data has been published for Belize. The latest WHO and UNICEF JMP hygiene data specific to Belize were from 2013 [6]. In 2009 and 2011, UNICEF Belize and the Belize Ministry of Education, Culture, Science, and Technology (MoECST) collaborated on an assessment of WASH resources at schools, but no contemporary assessments have since been conducted in Belizean schools [11].

The lack of recent national and systematic assessments of WASH infrastructure poses challenges for identifying gaps and informing public health decisions, especially during times of public health emergencies. During the COVID-19 pandemic, we conducted a national assessment of WASH infrastructure and resources in Belizean schools prior to the national reopening of schools for in-person learning. In-depth onsite assessments were conducted at 12 pilot schools shortly following the reopening of schools (baseline) and again 15 months later (follow-up). As the pandemic progressed, there may have been changes to the availability of WASH supplies at schools due to the public’s perceived risk, donations of supplies, and the economic circumstances of the local community. In this paper, we aim to document WASH infrastructure at schools and assess changes over two different time points during the pandemic.

## 2. Methods

### 2.1. National Survey

From December 2021 to January 2022, an electronic survey was distributed to all primary and secondary government and government-aided schools in Belize by the MoECST (N = 308). Among primary and secondary schools in Belize, 82% are operated by or receive support from the government, while the remaining are private. The survey questions covered school characteristics, access to water and hand hygiene resources, perceived water quality, access to toilets/latrines, and management of hand hygiene resources. The national survey questions were designed with reference to the JMP questions and indicators for monitoring WASH in schools [12]. The survey was to be completed by a school administrator or a staff member knowledgeable about the school’s policies and resources. The survey was only available in English and took approximately 20 min to complete. Survey responses were recorded on REDCap hosted at Baylor College of Medicine (BCM) [13,14].

### 2.2. Pilot School Selection

Since the pilot schools in this analysis were part of a larger study to pilot a hand hygiene intervention, a scoring system was developed to select these schools. Based on the responses from those schools completing the national survey, we compiled a list of schools with at least one of the following criteria: (1) did not have a functional handwashing station, and/or (2) did not have alcohol-based hand rub (ABHR). Since the in-depth evaluations were planned to be conducted in person, schools that were closed or only operating remotely were excluded. From the condensed list of schools, a scoring system was developed based on gaps in hand hygiene resources (Table 1). The score for each item was designed based on the importance of the resource and its relevance to the scope of the project. Higher scores indicated higher needs for hand hygiene resources at the schools. To understand potential variation in access to WASH services across Belize, two schools with the highest scores in each district were selected to serve as pilot schools for in-depth assessments and intervention. If multiple schools had the same score, the school with the higher student population was selected to maximize the impact of the intervention. Twelve schools were selected, two per district, for the pilot intervention.

### 2.3. Facility Assessment at 12 Pilot Schools

The baseline facility assessments were conducted in March 2022, and the follow-up facility assessments were conducted in June 2023. This timeframe was followed to allow time for the full development and implementation of the behavior change intervention at the pilot schools. Facility assessments were conducted by trained enumerators from BCM and CDC, with data entered into REDCap. Each facility assessment documented the source of water used at the school, the availability and functionality of water access points and ABHR dispensers on the school compound, and the availability of hand hygiene supplies in or near restrooms. A water access point was defined as a point where water can be reached for usage, such as a tap from a pipe or a water container. At each water access point, the enumerators checked and documented the type, use, and functionality of the access point (if water is present and the tap is mechanically working if there is one), the presence of soap and paper towels at access points used for handwashing, and the accessibility for the smallest children at the school and individuals with disabilities. The accessibility questions were based on the JMP criteria [12]. To be accessible for individuals with a disability, the water access point must be (1) accessible via a clear path, without stairs or steps, free of obstructions, (2) reachable from a seated position, and (3) usable with minimal effort with one closed fist or foot. A water access point that is accessible for small children is one that (1) can be reached by a small child and (2) can be easily used by small children. 

For each ABHR dispenser that was available for use, the enumerators documented the location, type, functionality (presence of ABHR inside), and size of the dispenser. We did not assess surplus ABHR in storage areas, nor did we assess personal ABHR dispensers brought by students or school staff for their use. For each school, the enumerators documented the total number of restrooms and the number of restrooms with a handwashing station with or without soap within five meters. 

### 2.4. Intervention at 12 Pilot Schools

The behavior change intervention period was from October 2022 to May 2023. Based on needs observed and shared by school staff during the baseline assessments, the schools were provided with liquid handwashing soap during the intervention to supplement their existing supplies (distributed in October 2022, March 2023, and May 2023). The amount of soap provided was proportional to the student population of the school assuming that each student uses approximately 3 mL of soap for handwashing per day based on a similar pilot WASH project in Guatemalan schools. Some schools were provided with bar soap. The pilot schools were instructed to place the soap by handwashing stations in the restrooms or handwashing stations that students usually use after leaving the restrooms. Environmental nudges (footpath from toilet to handwashing station or handwashing stickers behind stall, handwashing message above handwashing station, and arrow point to soap dispenser) and a hand hygiene workshop for school principals to design student hand hygiene lessons were also implemented. Schools were periodically monitored in person by local staff employed by BCM and community health workers from the Belize Ministry of Health and Wellness. During these monitoring visits, school administrators were reminded by the BCM staff and community health workers to place soap by the handwashing stations near the restrooms. 

### 2.5. Statistical Analyses

Descriptive statistics were calculated for the aggregate national survey data and the facility assessment data from baseline and follow-up. Percent changes were calculated for facility assessment items related to handwashing stations, ABHR dispensers, and restrooms with hand hygiene resources to evaluate changes between baseline and follow-up at the 12 pilot schools. The Chi-square test was used to compare the availability of WASH infrastructure and supplies at baseline and follow-up at the pilot schools. Statistical tests were considered significant at the 0.05 level. Analyses were performed using Stata version 17 [15]. 

## 3. Results

### 3.1. National Survey

Of the 308 government and government-aided primary and secondary schools registered with the MoECST in the 2021–2022 school year, 221 schools (72%) completed the national survey. There was participation from all six districts of Belize (Table 2). Most of the schools that responded to the national survey were rural (n = 146, 66%) and classified as primary schools (n = 191, 86%). There were differences in the distribution of schools that did and did not complete the national survey based on district and grade level. The number of students enrolled at schools that completed the national survey ranged from 5 to 1020 students. At the time the survey was conducted, most schools were still closed due to the COVID-19 pandemic (n = 137, 62%). All 12 schools selected for the intervention were primary schools, and 10 of the 12 were classified as rural. The number of students enrolled at the pilot schools ranged from 56 to 402 students. 

**Figure 1:**
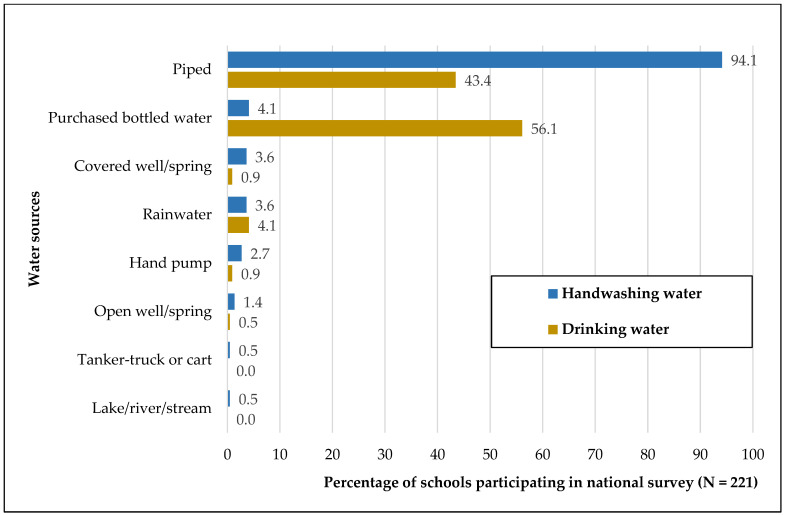
Sources of handwashing and drinking water reported by schools in the national survey *. * Responses are not mutually exclusive.

In the national survey, most schools reported using piped water for handwashing (n = 208, 94%) (Figure 1). Almost all urban schools (99%) reported using piped water for handwashing compared to 92% of rural schools. The most common source of drinking water was purchased bottled water (n = 124, 56%), followed by piped water (n = 96, 43%). Among urban schools, 88% used purchased bottled water, and 43% used piped water for drinking. In comparison, 40% of rural schools used purchased bottled water, and 44% used piped water for drinking. Other sources of drinking water at rural schools included covered wells/springs, rainwater, hand pumps, and open wells/springs. 

Approximately half the schools in the national survey reported that, at times, water was not available from the main water source at their school (n = 121, 55%) (Table 3). Nearly one-third of schools expressed concerns about the quality of the water at their school (n = 65, 29%), and the most common concerns were the taste and color of the water. 

Almost all schools reported having a handwashing station (n = 219, 99%), and a high percentage reported having a functional handwashing station (n = 197, 89%) (Table 3). Nearly half the schools (n = 103, 47%) reported always having soap for handwashing, but a lower proportion of schools (n = 45, 20%) reported always having ABHR for student use. Almost all schools reported having restrooms that are separated by sex (n = 210, 95%). A flush toilet was the most common (n = 200, 91%) type of toilet reported. 

### 3.2. Pilot School Assessment

The facility assessment was conducted at all 12 pilot schools at baseline and at 11 of the 12 pilot schools at follow-up since one school was not operating under normal conditions at the time of the follow-up assessment. There was a decrease in the proportion of functional handwashing access points between baseline and follow-up, from 91% to 80% (*p* = 0.007) (Table 4). The percentage of functional handwashing access points with soap present was similar between baseline and follow-up, 74% and 73%, respectively (*p* = 0.828). The percentages of functional handwashing stations accessible for individuals with disabilities and for small children decreased from baseline to follow-up (61% to 44%, *p* = 0.007 and 84% to 55%, *p* < 0.001, respectively). In a sensitivity analysis excluding the one school that was not operating under normal conditions at follow-up, there was no significant difference in the proportion of functional handwashing access points accessible for individuals with disabilities (Table S1). Nearly all (97%) of ABHR dispensers were functional at the time of the baseline assessment compared with 84% at follow-up (*p* = 0.001). In aggregate, the presence of hand hygiene resources within 5 m of the restroom did not vary between baseline and follow-up. However, at baseline, all pilot schools had at least one restroom with a handwashing station with water and soap within 5 m compared to 9 schools at follow-up. 

## 4. Discussion

In a national survey of 221 schools in Belize, schools reported challenges with water availability and quality. Additionally, constant access to soap for handwashing was a gap reported. The in-person assessment of a subset of 12 pilot schools in March 2022 (baseline) and June 2023 (follow-up) showed a decrease in the proportion of handwashing access points that were functional and accessible for individuals with disabilities and small children. 

We hypothesize that these observed decreases in hand hygiene resources between baseline and follow-up were related to the timing of the assessments in relation to the COVID-19 pandemic, with mandates and vigilance for hand hygiene and disease prevention efforts being heightened at the time of the baseline assessment. Many changes in COVID-19 protocols and mandates occurred over the course of this study. The national survey was conducted when many schools were still closed due to the COVID-19 pandemic in December 2021 or immediately before schools reopened in early January 2022. However, the in-person baseline assessments at the pilot schools were conducted in March 2022, shortly after most schools reopened for in-person learning [16]. To reopen, schools were required to meet the criteria set forth by the MoECST, many of which were related to hand hygiene. For example, schools were required to have a handwashing or sanitizing station at the entrance for students to clean their hands as they entered school grounds. As a result, there may have been changes to hand hygiene infrastructure and resources between the timing of the national survey and the baseline assessments. In the early months of the school reopening, the MoECST also distributed hygiene kits (disinfectants, soap, ABHR, masks, etc.) upon school request (meeting with Y. Gongora, March 2022), which may have potentially increased the availability of supplies during the baseline assessment.

The national survey and the baseline assessment were conducted amidst the COVID-19 pandemic before the COVID-19 vaccine was available to primary-school-age children in Belize [17]. Furthermore, the baseline assessment was conducted soon after Belize had experienced the largest wave of COVID-19 cases [18]. However, the follow-up assessment was conducted after the WHO had declared that COVID-19 was no longer a public health emergency of international concern [19]. At the time of the follow-up assessment, many precautionary measures were no longer mandated in school settings in Belize (such as requiring students to wash hands or use ABHR upon entering school grounds). These changes may have influenced the awareness and perceived importance of hand hygiene, leading to a decrease in the prioritization of hand hygiene resources at schools. 

The gaps in WASH resources and infrastructure documented in this study are consistent with results from other studies. A systematic review of WASH in LMICs published in 2022 suggested that schools in other LMICs also consistently experienced gaps in access to clean water [20]. The proportion of schools reporting concerns with the quality of water is consistent with data from a national survey conducted in Belize in 2009 by UNICEF and the MoECST, which stated that one in four schools reported having untreated water [11]. Both the baseline and follow-up assessment at the pilot schools showed gaps in hand hygiene infrastructure access for small children and individuals with disabilities. This finding is similar to what was highlighted in the 2009 assessment, where only 70% of schools in Belize had water facilities accessible for small children and children with disabilities, and this proportion was as low as 62% in Belize District [11]. Accessible WASH infrastructure at schools is vital to ensuring an equitable educational environment for all, as outlined in the SDG; however, there is often a lack of standardized disability-inclusive data [21]. Our assessment included the standardized accessibility criteria from the JMP, which allows for comparison across multiple settings and countries and provides a baseline for assessing changes in access in the future. 

A lack of soap for handwashing was also observed in WASH assessments in other LMICs [20]. A previous survey demonstrated that almost 30% of schools in Belize did not have soap for handwashing [11,22]. At the time of our national survey, only 3% of schools reported never having soap for handwashing. During the COVID-19 pandemic, schools were required to have soap for handwashing; therefore, schools may have sought donations of hand hygiene supplies from parents or purchased supplies using their school funds. Some schools were able to apply to receive assistance from the MoECST. 

As part of the behavior change intervention, the 12 pilot schools received handwashing soap to supplement the schools’ supplies for the restrooms; however, at follow-up, we did not observe an improvement in soap presence at handwashing stations in or near the restrooms. This is most likely due to the management systems of hand hygiene supplies at the schools. Many schools preferred to keep soap inside the classrooms for students to take to the restroom instead of leaving the soap bottles by the restroom handwashing stations. School administrators shared that the students waste soap, and/or the soap dispensers sometimes get stolen if left unattended by the handwashing stations. 

To our knowledge, this study was the most recent one to conduct a comprehensive assessment of WASH infrastructure and resources in schools across all districts of Belize and the only study to assess WASH in schools in Belize during the COVID-19 pandemic. Many of the schools that participated were in rural areas of Belize. Additionally, the pilot schools selected also spanned all six districts of Belize and included schools with more WASH needs, allowing us to assess hand hygiene conditions in diverse, high-need settings. Although our pilot schools were from all six districts of Belize, the results may not be generalizable to other schools in Belize as there were a small number of pilot schools included in the full assessments, and their selection into this study depended on their participation in the national survey and having higher self-reported hand hygiene needs.

There are several other limitations to this study. Since the national survey was administered electronically, there may be a bias toward those schools with the resources to complete the survey (internet, electronic devices, time, etc.). With a self-administered questionnaire, it is possible that school staff misinterpreted some questions. Additionally, the self-reported information may not accurately represent the actual availability of infrastructure and resources. The schools participating in the national survey and those selected for the pilot evaluation and intervention may not be representative of all Belizean schools. For the follow-up evaluation, schools were aware of the purpose of the assessments and had already worked closely with BCM for over a year during the intervention period, which could have resulted in positive responses if some schools anticipated the follow-up assessments. Nevertheless, we observed a reduction in hand hygiene resources at follow-up. Although all enumerators were trained in the data collection process, there may be slight differences in the baseline and follow-up data due to different staff conducting the assessments. Due to unforeseen circumstances, one school was not operating under normal conditions at follow-up; therefore, it was excluded from the follow-up evaluation. 

Future hand hygiene interventions should consider each school’s operational, cultural, and social contexts and school preferences to ensure that hand hygiene resources are available and easily accessible. Standardized systems for WASH management, along with tailored intensive hand hygiene promotion campaigns, are important to ensure the availability and use of WASH resources in schools [23,24]. Routine assessments of WASH resources at a national and local level are critical to monitoring access and informing public health actions.

## 5. Conclusions

Access to WASH resources in school settings is vital to ensuring a healthy and effective learning environment for students. Our national assessment provided a comprehensive understanding of WASH in schools in Belize and served as a foundation for future WASH interventions to bridge the gaps in infrastructure and resources at schools in Belize. Continuous monitoring of WASH indicators is important for informing public health guidance and activities in community settings. 

## Figures and Tables

**Table 1:** The scoring system used to rank schools based on hand hygiene resource gaps.

Criterion	Score
**Handwashing station**	
No functional handwashing station	5
Functional handwashing station present	0
**Soap**	
No soap available	4
There is sometimes soap available	3
There is always soap available	0
**Alcohol-based hand rub (ABHR)**	
No ABHR available	3
There is sometimes ABHR available	2
There is always ABHR available	0
**Water**	
There are times when water is not available	2
Water is always available	0
There is a concern about water quality	1
There is no concern about water quality	0

**Table 2:** Comparison of characteristics among national survey non-participating and participating schools and pilot schools.

	Schools that Did not Complete the National Survey (N = 87) n (%)	Schools That Completed the National Survey (N = 221) n (%)	Pilot Schools Selected for Intervention (N = 12) n (%)
District **			
Corozal	3 (3)	44 (20)	2 (17)
Orange Walk	4 (5)	35 (16)	2 (17)
Belize	15 (17)	49 (22)	2 (17)
Cayo	35 (40)	34 (15)	2 (17)
Stann Creek	7 (8)	24 (11)	2 (17)
Toledo	23 (26)	35 (16)	2 (17)
**Locality**			
Rural	60 (69)	146 (66)	10 (83)
Urban	27 (31)	75 (34)	2 (17)
Grade level **			
Primary	67 (77)	191 (86)	12 (100)
Secondary	20 (23)	30 (14)	0 (0)
**School operation**			
Open/Hybrid	-	84 (38)	12 (100) *
Closed	-	137 (62)	0 (0)
**Number of classrooms, median (IQR)**	-	9 (10)	8.5 (9)
**Student enrollment, median (IQR)**	-	195 (246)	177 (185)

IQR = interquartile range. * All pilot schools were operating in hybrid mode at the time of the baseline assessments and were operating fully in person at the time of the follow-up assessment (with the exception of one pilot school that was not operating under normal conditions at follow-up). ** Statistical difference between national survey non-participating and participating schools at the 0.05 level.

**Table 3:** Water access and hand hygiene resources at schools detailed in the national survey.

Questions from the National Survey	N = 221 n (%)
**There are times when water is not available**	121 (55)
**Concerns about water quality**	65 (29)
Type of concern *	
Color of water	28 (13)
Smell of water	14 (6)
Taste of water	32 (14)
Contamination of water source	24 (11)
**There is a handwashing station**	219 (99)
**There is a functional handwashing station**	197 (89)
**There is soap available for handwashing**	
Always	103 (47)
Sometimes	110 (50)
Never	6 (3)
**There is alcohol-based hand rub available for students**	
Always	45 (20)
Sometimes	89 (40)
Never	87 (39)
**There are restrooms separated by sex**	210 (95)
**Most common type of toilet on school ground**	
Flush/pour-flush toilet	200 (91)
Pit latrines with slab	16 (7)
Pit latrines without a slab	2 (1)

* Responses are not mutually exclusive.

**Table 4:** Hand hygiene infrastructure at 12 pilot schools * at baseline and follow-up.

	Baseline	Follow-Up	Percent Change	*p*-Value
Handwashing Station				
**Total handwashing access points**	165	142	-	-
Functional handwashing access points **	150 (91%)	114 (80%)	−11%	**0.007**
Functional handwashing access points with soap, n (%) ^†^	111 (74%)	83 (73%)	−1%	0.828
Functional handwashing access points with paper towels, n (%) ^†^	63 (42%)	50 (44%)	+2%	0.762
Functional handwashing access points accessible for individuals with disabilities, n (%) ^†^	91 (61%)	50 (44%)	−17%	**0.007**
Functional handwashing access points accessible for small children, n (%) ^†^	126 (84%)	63 (55%)	−29%	**<0.001**
**ABHR**				
**Total ABHR dispensers**	119	94		
Functional ABHR dispensers	115 (97%)	79 (84%)	−13%	**0.001**
**Restrooms**				
**Total restrooms**	61	53		
Restrooms with a handwashing station with water within 5 m, n (%)	53 (87%)	39 (74%)	−13%	0.073
Restrooms with a handwashing station with water and soap within 5 m, n (%)	42 (69%)	35 (66%)	−3%	0.749

ABHR = alcohol-based hand rub. * Twelve pilot schools assessed at baseline; 11/12 pilot schools assessed at follow-up. ** Percent of all handwashing access points. ^†^ Percent of all functional handwashing access points. Statistical significance at 0.05 level is denoted in bold.

## Data Availability

All relevant data are included in this article.

## Supplementary Materials

The following supporting information can be downloaded at https://www.mdpi.com/article/10.3390/ijerph21040470/s1; Table S1: Hand hygiene infrastructure at 11 pilot schools at baseline and follow-up *.
