# Supplementary material for: Water, Sanitation, and Hygiene Infrastructure and Resources in Schools in Belize during the COVID-19 Pandemic, 2021–2023
Source: Int J Environ Res Public Health. 2024 Apr 12;21(4):470. doi: 10.3390/ijerph21040470 (PMC11050648; doi:10.3390/ijerph21040470)
Supplement: Supplementary file 1 [file ijerph-21-00470-s001.zip › ijerph-2948008-supplementary/ijerph-2948008-SI-main.pdf]

**Table S1.** Hand hygiene infrastructure at 11 pilot schools at baseline and follow-up*.

| | Baseline | Follow-up | Percent change | P-value |
|---|---|---|---|---|
| **Handwashing station** | | | | |
| **Total handwashing access points** | 146 | 142 | - | - |
| **Functional handwashing access points**** | 131 (90%) | 114 (80%) | -10% | **0.025** |
| Functional handwashing access points with soap, n (%)† | 94 (72%) | 83 (73%) | +1% | 0.855 |
| Functional handwashing access points with paper towel, n (%)† | 50 (38%) | 50 (44%) | +6% | 0.366 |
| Functional handwashing access points accessible for individuals with disabilities, n (%)† | 72 (55%) | 50 (44%) | -11% | 0.083 |
| Functional handwashing access points accessible for small children, n (%)† | 108 (82%) | 63 (55%) | -27% | **<0.001** |
| **ABHR** | | | | |
| **Total ABHR dispensers** | 106 | 94 | | |
| Functional ABHR dispensers | 102 (96%) | 79 (84%) | -12% | **0.003** |
| **Restrooms** | | | | |
| **Total restrooms** | 52 | 53 | | |
| Restrooms with handwashing station with water within 5 meters, n (%) | 45 (87%) | 39 (74%) | -13% | 0.097 |
| Restrooms with handwashing station with water and soap within 5 meters, n (%) | 34 (65%) | 35 (66%) | +1% | 0.944 |

ABHR = alcohol-based hand rub

*Excluded one school that was not operating in normal conditions at follow-up

**Percent of all handwashing access points

†Percent of all functional handwashing access points
