# Supplementary material for: Water, Sanitation, and Hygiene Infrastructure and Resources in Schools in Belize during the COVID-19 Pandemic, 2021–2023
Source: Int J Environ Res Public Health. 2024 Apr 12;21(4):470. doi: 10.3390/ijerph21040470 (PMC11050648; doi:10.3390/ijerph21040470)
Supplement: Supplementary file 1 [file ijerph-21-00470-s001.zip › ijerph-2948008-supplementary/ijerph-2948008-supplementary.pdf]

National School WASH Survey

School Information

In this section, we will ask general questions about your school location and your role at the school.

Today's date

____

What is the name of your school?

____

In which district is your school located?

- ◯ Corozal
- ◯ Orange Walk
- ◯ Belize
- ◯ Cayo
- ◯ Stann Creek
- ◯ Toledo

In which city, town, or village is your school located?

____

What kind of school do you work in?

- ◯ Primary
- ◯ Secondary
- ◯ Other

If other, please specify your school type.

____

What is your name?

____

What is your position in the school?

- ◯ Principal/administrator
- ◯ Teacher
- ◯ Other

If other, please specify your position at your school.

____

School Demographics

In this section, we will ask questions about your school structure and function.

Total students enrolled in your school: ______________________

Since March 2020, has your school reopened for face-to-face attendance?

- ◯ Yes
- ◯ No

How many times has it been closed because of COVID-19?

- ◯ None
- ◯ Once
- ◯ Twice
- ◯ More than two times

Is your school currently closed as a result of COVID-19?

- ◯ School is open
- ◯ School is closed
- ◯ Operating in hybrid mode

In the past two weeks, how many students on average attended face-to-face classes per day? ______________________

In the past two weeks, how many students on average attended online classes or were learning remotely per day? ______________________

How many students do you anticipate returning to the classroom when school reopens? ______________________

How many students do you anticipate attending classes online or learning remotely when school reopens? ______________________

How many teachers are currently teaching face-to-face? ______________________

How many classrooms are there in your school (separated by a wall)? ______________________

Does your school provide food to students through a school feeding program?

Select all that apply.

- ☐ Yes, students eat food cooked on-site
- ☐ Yes, students eat food we purchase from an outside vendor
- ☐ Yes, but students also have the option to bring food from home
- ☐ No school food program. Students leave the campus to eat lunch
- ☐ No school food program. Students bring food from home
- ☐ Other

If other, please describe your school feeding program. ______________________

Will your school provide food to students through a school feeding program when it reopens?<br><br>Select all that apply.	☐ Yes, students will eat food cooked on-site<br>☐ Yes, students will eat food we purchase from an outside vendor<br>☐ Yes, but students will also have the option to bring food from home<br>☐ No school food program. Students will leave the campus to eat lunch<br>☐ No school food program. Students will bring food from home<br>☐ Other
If other, please describe your school feeding program.	______________________________
Where do students currently eat?<br><br>Select all that apply.	☐ Indoors in their classrooms<br>☐ Indoors in a school cafeteria or dining hall<br>☐ Outdoors<br>☐ At home or away from the school<br>☐ Other
If other, please specify where students eat.	______________________________
Where will students eat when they return to the classroom?<br><br>Select all that apply.	☐ Indoors in their classrooms<br>☐ Indoors in a school cafeteria or dining hall<br>☐ Outdoors<br>☐ At home or away from the school<br>☐ Other
If other, please specify where students eat.	______________________________

Water Access

In this section, we will ask questions about the school's main water source and restrooms.

What is the main source of water of the school?<br><br>Select the one used most frequently.	◯ Piped<br>◯ Covered well/spring<br>◯ Open well/spring<br>◯ Manual hand pump<br>◯ Rainwater<br>◯ Purchased bottled water<br>◯ Tanker-truck or cart<br>◯ Lake/river/stream<br>◯ No water source<br>◯ Other<br>◯ Don't know
If other, please specify the main source of water at the school.	____________________
Where do you access the main water source for this school?	◯ On school ground<br>◯ Off school ground
How long does it take to go to the main source, collect water, and return?	◯ Less than an hour<br>◯ 1-2 hours<br>◯ More than 2 hours
Are there ever times when water is not available at this source?	◯ Yes<br>◯ No<br>◯ Don't know
What are the reasons that water is sometimes not available?<br><br>Select all that apply.	☐ Temporary / daily shortage (low water table, etc.)<br>☐ Locked / restricted access<br>☐ Power outages<br>☐ Seasonal shortage<br>☐ Inability to pay / too expensive<br>☐ Mechanical breakdown<br>☐ Other
If other, please specify why water is sometimes not available.	____________________
Are there ever concerns about the quality or cleanliness of water at the school?	◯ Yes<br>◯ No
What is the concern about the water?<br><br>Select all that apply.	☐ Color of water<br>☐ Smell of water<br>☐ Taste of water<br>☐ Contamination of water source<br>☐ Other
If other, please specify your concern about the water.	____________________

What is the source of water for handwashing?<br><br>Select all that apply.	☐ Piped<br>☐ Covered well/spring<br>☐ Open well/spring<br>☐ Manual hand pump<br>☐ Rainwater<br>☐ Purchased bottled water<br>☐ Tanker-truck or cart<br>☐ Lake/river/stream<br>☐ No water source<br>☐ Other<br>☐ Don't know
If other, please specify the water source for handwashing.	______________________
Is there drinking water at the school?	◯ Yes<br>◯ No
What is the source of drinking water?<br><br>Select all that apply.	☐ Piped<br>☐ Covered well/spring<br>☐ Open well/spring<br>☐ Manual hand pump<br>☐ Rain water<br>☐ Purchased bottled water<br>☐ Tanker-truck or cart<br>☐ Lake/river/stream<br>☐ Other<br>☐ Don't know
If other, please specify the drinking water source.	______________________
Why is there no drinking water available at school?<br><br>Select all that apply.	☐ Concerns about quality of water<br>☐ Distance to water source too far<br>☐ Water sometimes not available at source (broken, seasonal shortage, etc.)<br>☐ No time to collect water<br>☐ No containers for water<br>☐ Water containers were broken or stolen<br>☐ No teacher to take responsibility<br>☐ No students to take responsibility<br>☐ No funds / not affordable<br>☐ Other
If other, please specify why there is no drinking water at the school.	______________________
How many restrooms are on the school grounds?	______________________
Are the restrooms separated by sex?	◯ Yes<br>◯ No
How many toilets/latrines are on the school grounds?	______________________

What type of toilets/latrines are at the school?<br><br>Select the most common option.	◯ Flush/pour-flush toilet<br>◯ Pit latrines with slab<br>◯ Pit latrines without slab<br>◯ Other
If other, please specify what type of toilet/latrine is available at your school.	______________________________

Hand Hygiene

In this section, we will ask questions about handwashing and hand sanitizer stations at your school.

Does the school have handwashing stations?	◯ Yes<br>◯ No
How many handwashing stations are there in total?	________________________
How many handwashing stations are not functioning?	________________________
Where are these handwashing stations located?<br><br>Select all that apply.	☐ At or near the entrance of the school<br>☐ Restrooms<br>☐ Food preparation area<br>☐ Food consumption area<br>☐ Classroom<br>☐ School yard<br>☐ Other
If other, please specify where the handwashing stations are located.	________________________
What type of handwashing stations are available?<br><br>Select all that apply.	☐ Fixed sink with water faucet<br>☐ A barrel or container with a spicket/tap<br>☐ A container<br>☐ A pila/basin<br>☐ Other
If other, please specify the type of handwashing station available.	________________________
Does the school have soap for handwashing?	◯ Yes, sometimes<br>◯ Yes, always<br>◯ No, never
How many handwashing stations currently have soap?	________________________
Why does the school not have soap for handwashing?<br><br>Select all that apply.	☐ No funds<br>☐ Soap is too expensive<br>☐ Soap gets used up too quickly<br>☐ Soap gets stolen<br>☐ Only provided when water is available<br>☐ Other
If other, please specify why the school does not have soap for handwashing.	________________________
Does the school have hand sanitizer available to students?	◯ Yes, sometimes<br>◯ Yes, always<br>◯ No, never

Why does the school not have hand sanitizer?	☐ No dispensers<br>☐ Dispensers are broken or stolen<br>☐ No time to fill dispensers<br>☐ No one to take responsibility<br>☐ Not affordable<br>☐ Not needed<br>☐ Other
If other, please specify why does the school not have hand sanitizer.	________________________
How many hand sanitizer dispensers are at the school (including bottle dispensers)?	________________________
How many hand sanitizer dispensers are not functioning?	________________________
What type of dispensers are used?<br><br>Select all that apply.	☐ Fixed dispenser to the wall that is battery operated/automated with hand sensors<br>☐ Fixed dispenser to the wall that you push to dispense<br>☐ Plastic bottle or container with pump<br>☐ Spray bottle<br>☐ Foot lever system<br>☐ Other
If other, please specify the type of dispensers.	________________________
Where are the hand sanitizer stations located?<br><br>Select all that apply.	☐ At the entrance of the school<br>☐ Next to the bathrooms<br>☐ In the hallways<br>☐ In the classrooms<br>☐ At classroom entrances<br>☐ In the cafeteria or dining hall<br>☐ Staff lounge<br>☐ Other
If other, please specify where the hand sanitizer stations are located.	________________________
Who provides the hand sanitizer to the school?<br><br>Select all that apply.	☐ Government of Belize<br>☐ Teachers<br>☐ Parents<br>☐ Non-profit organizations<br>☐ Other<br>☐ Don't know
If other, please specify who provides the hand sanitizer to the school.	________________________
Is the amount of hand sanitizer provided sufficient?	◯ Yes<br>◯ No<br>◯ Don't know

**School Management of Hygiene Services**<br><br>**In this section, we will ask questions about expenses and funding for school reopening.**	
What specific areas have funds been spent in order to have the school qualified to safely reopen during COVID-19? (HWS = handwashing station)<br><br>Select all that apply.	☐ Water or water delivery<br>☐ Adding new HWS<br>☐ Repairs of HWS<br>☐ Soap for handwashing<br>☐ Cleaning supplies (mops, brooms, detergent, etc.)<br>☐ Purchasing or repairing water containers<br>☐ Building, purchasing or repairing container stands<br>☐ Repairs of restrooms/toilets<br>☐ Construction of restrooms/toilets<br>☐ Purchasing hand sanitizer<br>☐ Purchasing dispensers for hand sanitizer<br>☐ Purchasing toilet paper<br>☐ Training materials for teachers<br>☐ Educational materials for students and families<br>☐ Signs and posters about hand hygiene<br>☐ No funds were spent<br>☐ Other
If other, please specify areas where funds have been spent for school reopening.	______________________________
Where did the funds come from?<br><br>Select all that apply.	☐ Government of Belize<br>☐ Donation from parents<br>☐ Donation from teachers<br>☐ Donations from non-profits<br>☐ Donations from religious organizations<br>☐ Other<br>☐ Don't know
If other, please specify where the funds came from.	______________________________
Which organization(s)?	______________________________
In addition to funds, what other type of assistance did the school receive from the above organization(s)?<br><br>Select all that apply.	☐ None<br>☐ Donation of handwashing stations<br>☐ Donation of soap<br>☐ Donation of hand sanitizer<br>☐ Training materials for teachers<br>☐ Educational materials for students and families<br>☐ Signs and posters about hand hygiene<br>☐ Other
If other, please specify type of assistance.	______________________________

Facility Assessment: Water Access Point

Please complete the form below for each water access point at the school.

Location of assessment	◯ Entrance<br>◯ Hallway<br>◯ Classroom<br>◯ Bathroom<br>◯ Dining area<br>◯ Staff room<br>◯ Other
If other, please specify location of assessment.	______________________________
What type of water access point is this?	◯ Pipe tap<br>◯ Portable container (bucket, jerrycan, etc.)<br>◯ Stationary container (cement tank, plastic drums, etc.)<br>◯ Other water source (borehole, well, etc.)<br>◯ Pila<br>◯ Drinking fountain<br>◯ Other
If other, please specify type of water access point.	______________________________
What is this water point used for?<br><br>Select all that apply.	☐ Drinking<br>☐ Handwashing<br>☐ Flushing toilets<br>☐ Cleaning<br>☐ Other
If other, please specify what the water point is used for.	______________________________
Is this water source for students or staff?	◯ Students<br>◯ Staff<br>◯ Both students and staff
Is there a lid in place?	◯ Yes<br>◯ No<br>◯ Not applicable
Is there a tap?	◯ Yes<br>◯ No
Is the tap functional?<br><br>Please use source to verify.	◯ Yes<br>◯ No<br>◯ Cannot assess (no water)
Is the water point leaking or broken?	◯ Yes<br>◯ No<br>◯ Cannot assess (no water)
Is water available for use right now?	◯ Yes<br>◯ No

Is soap available?	◯ Yes<br>◯ No
Is paper towel available?	◯ Yes<br>◯ No
Is this DRINKING WATER access point accessible by people with disabilities?<br><br>1. Accessible via a clear path without stairs or steps that is free of obstructions<br>2. Can be reached from a seated position<br>3. The water source/dispenser can be used with minimal effort with one closed fist or feet	◯ Yes<br>◯ No
Is this DRINKING WATER access point accessible by the smallest children in the school?<br><br>1. Can be reached by a small child<br>2. The water source/dispenser can be used by small children	◯ Yes<br>◯ No
Is this HANDWASHING access point accessible by people with disabilities?<br><br>1. Accessible via a clear path without stairs or steps that is free of obstructions<br>2. Can be reached from a seated position<br>3. The water source/dispenser can be opened/closed with minimal effort with one closed fist or feet	◯ Yes<br>◯ No
Is this HANDWASHING access point that can be accessed by the smallest children in the school?<br><br>1. Can be reached by a small child<br>2. The water source/dispenser can be opened/closed by small children	◯ Yes<br>◯ No

Facility Assessment: Alcohol-Based Hand Rub

Please complete the form below for each hand sanitizer dispenser (including spray bottles) at the school.

Date

____

Location of assessment

- ◯ School entrance
- ◯ Hallway
- ◯ Inside classroom
- ◯ Classroom entrance
- ◯ Bathroom
- ◯ Dining area
- ◯ Staff room
- ◯ Other

If other, please specify location of assessment.

____

What type of dispenser is this?

- ◯ Spray bottle
- ◯ Pump top bottle
- ◯ Push-style
- ◯ Automatic
- ◯ Foot pedal
- ◯ Other

If other, please specify dispenser type.

____

How is the dispenser stationed?

- ◯ No fixed location (countertop, tables, etc.)
- ◯ Fixed location (wall)
- ◯ Self-standing (floor)
- ◯ Other

If other, please specify how the dispenser is stationed.

____

What is the volume of the dispenser (in mL)?

- ◯ Less than 500 mL
- ◯ About or greater than 500mL

Is there currently alcohol handrub in the dispenser?

Please use the dispenser to verify.

- ◯ Yes
- ◯ No
- ◯ Cannot observe dispenser closely to assess alcohol presence
- ◯ Dispenser is broken so cannot assess alcohol presence

How often is the dispenser empty?

- ◯ Rarely
- ◯ Sometimes
- ◯ Always
- ◯ Do not know

Is the dispenser broken?

Please use the dispenser to verify.

- ◯ Yes
- ◯ No
- ◯ Cannot test to verify

How long has the dispenser been broken?	◯ Less than a week<br>◯ More than a week but less than a month<br>◯ More than a month<br>◯ Do not know
Is the dispenser refillable or does it use replacement cartridges?	◯ Refillable<br>◯ Replace container<br>◯ Other<br>◯ Do not know
If other, please specify.	______________________________
How many times per month is this dispenser typically refilled?	◯ 10 or more times per month (every few days)<br>◯ 5-9 times per month<br>◯ 1-4 times per month<br>◯ Less than 1 time per month Have<br>◯ never refilled<br>◯ Do not know

Facility Assessment: Restrooms

Please complete the form below. Only one form per school.

A restroom is defined as a room that contains one or more toilets/latrines/stalls. If the male and female sides are completely separated by a wall (each with one or more toilets/latrines/stalls inside) then we will count the male side as one restroom and female side as another restroom (2 total).

1) Date ______________________________

2) How many restrooms are there? ______________________________

3) How many restrooms are for male students? ______________________________

4) How many restrooms are for female students? ______________________________

5) How many restrooms are for staff? ______________________________

For the next two questions, the handwashing station or hand sanitizer dispenser can be inside the restroom or within 5 meters of the restroom.

6) How many of the restrooms have a handwashing station with water? ______________________________

7) How many of the restrooms have a handwashing station with water and soap? ______________________________

8) How many of the restrooms have a functional hand sanitizer dispenser outside? ______________________________
